# Understanding dynamic complexity in context—Enriching contextual analysis in implementation science from a constructivist perspective

**DOI:** 10.3389/frhs.2022.953731

**Published:** 2022-07-22

**Authors:** Juliane Mielke, Sabina De Geest, Franziska Zúñiga, Thekla Brunkert, Leah L. Zullig, Lisa M. Pfadenhauer, Sandra Staudacher

**Affiliations:** ^1^Institute of Nursing Science, Department Public Health, University of Basel, Basel, Switzerland; ^2^Academic Center for Nursing and Midwifery, Department of Public Health and Primary Care, KU Leuven, Leuven, Belgium; ^3^University Department of Geriatric Medicine FELIX PLATTER, Basel, Switzerland; ^4^Center for Innovation to Accelerate Discovery and Practice Transformation (ADAPT), Durham Veterans Affairs Health Care, Durham, NC, United States; ^5^System and Department of Population Health Sciences, School of Medicine, Duke University, Durham, NC, United States; ^6^Institute for Medical Information Processing, Biometry and Epidemiology, Ludwig Maximilian University of Munich, Munich, Germany; ^7^Pettenkofer School of Public Health, Ludwig Maximilian University of Munich, Munich, Germany; ^8^Department of Health Services Research, Care and Public Health Research Institute, Maastricht University, Maastricht, Netherlands

**Keywords:** context, implementation science, dissemination, social science, constructivism, post-positivism, contextual analysis, complexity

## Abstract

Context in implementation science includes not only characteristics of a setting in which an intervention will be delivered, but also social systems (e.g., interrelationships). Context is dynamic and interacts with both, the intervention and its implementation. Therefore, contextual analysis is recognized as an indispensable part of implementation science methodology: it provides the foundation for successful and sustainable implementation projects. Yet, driven by the prevailing post-positivist understanding of context, contextual analysis typically focuses on individual characteristics of context i.e., contextual dynamics and interactions go unnoticed. Conducting contextual analysis from a constructivist perspective promotes a multilayered approach, building a more comprehensive understanding of context, and thus facilitating successful implementation. In this article, we highlight the limitations of prevailing perspectives on context and approaches to contextual analysis. We then describe how contextual analysis can be enriched by working from a constructivist perspective. We finish with a discussion of the methodological and practical implications the proposed changes would entail. Emerging literature attempts to address both the concept of context and methods for contextual analysis. Various theories, models and frameworks consider context, however, many of these are reductionistic and do not acknowledge the dynamic nature of context or interactions within it. To complement recent conceptualizations of context, we suggest consider the following five constructivist concepts: 1) social space; 2) social place; 3) agency; 4) sensation; and 5) embodiment. We demonstrate the value of these concepts using COVID-19 vaccination uptake as an example and integrate the concepts in the *Context and Implementation of Complex Interventions (CICI) framework—*an implementation science framework that pays ample attention to context. To study context from a constructivist perspective, we also suggest additional considerations in view of methodologies for data collection and analysis, e.g., rapid ethnographic methods. A constructivist perspective contributes to a stronger conceptualization of contextual analysis. Considering the five constructivist concepts helps to overcome contextual analysis' current shortcomings, while revealing complex dynamics that usually go unnoticed. Thus, more comprehensive understanding of context can be developed to inform subsequent phases of an implementation project, thereby maximizing an intervention's uptake and sustainability.

## Introduction

Contextual analysis is a foundational phase within the implementation science (IS) methodology, and essential to successful and sustainable implementation of interventions in real-world settings (1, 2). To our knowledge, there is no standard definition of contextual analysis. We define contextual analysis as a distinct aspect of an IS project that begins prior to when an intervention is developed/adapted and implemented. Contextual analysis typically includes a theory-supported mapping of a range of relevant aspects in context (often labeled as facilitators/barriers). Context in IS includes not only characteristics of a setting in which an intervention will be delivered, but also social systems (e.g., interrelationships) (3, 4). Context is dynamic and interacts with both, the core components of an intervention and its implementation (4). Therefore, understanding context is key, to inform all subsequent phases of an IS study, i.e., intervention development/adaptation (1, 5–7), choices of implementation strategies (8–10), interpretation of implementation and effectiveness outcomes (6, 10–12), choice of sustainability strategies (12) and scale-up (12, 13). Given that context evolves over time, repeated assessments of context should be conducted throughout the project.

While increasingly facilitators and barriers to implementation success are being mapped—often not theory based—contextual analyses are often performed isolated from their IS projects' next phases (2, 14, 15). This reflects the implicit post-positivist assumption that context is a static background. Based on this assumption, contextual analysis commonly focuses on individual characteristics of context without considering dynamic interactions (16, 17). This mindset hampers both the tailoring of interventions to target contexts and the selection of contextually adapted implementation strategies, limiting the ultimate goal of IS to enhance implementation success (18).

Shaped by public health, education, social work, environmental science, and political science, among others, IS has gained traction over the past two decades with researchers in evidence-based medicine and public health (19, 20). As a field of research, IS achieved considerable theoretical and methodological advances, developed a variety of theories, models and frameworks (TMFs) (21, 22), applied rigorous methods e.g., to assess implementation processes, mechanisms and outcomes (23–25), and developed measurement tools to analyze context (26). A number of these advancements are strongly connected with a post-positivist understanding of context.

The post-positivist paradigm is cause-and-effect oriented, recognizing “*all cause and effect [as] a probability that may or may not occur*” [(27), p. 59]. By that, only artifacts, i.e., individual aspects of context are studied; relationships of aspects in context to each other, their underlying structures, values, beliefs and culture, are usually dismissed—all of which limits a holistic understanding of context as a complex and dynamic system (28). Implementation takes place in social contexts where implementation agents, the context, intervention and implementation all interact continuously (16). Implementation agents (hereinafter referred to as agents) include three main groups of individuals or organizations: those directly targeted or affected by an intervention (e.g., patients and their relatives); those that decide on that intervention's implementation (e.g., leaders, politicians, funders); and those that implement the intervention (e.g., healthcare providers) (4). These agents' actions are based on their beliefs, norms, values, and identities, all of which are shaped by the contexts in which they are located. Likewise, through their actions, agents can shape and alter their context. Therefore, as part of the contextual analysis, it is crucial to understand how agents are embedded in a context, how the context influences their actions, and how they can shape and reshape the context. Naturally, any changes to the context also influence the implementation.

A paradigm, that acknowledges dynamic interactions in context, based on the lived experiences of agents within context, is constructivism [(27), p.60]. We assert that enriching the prevailing post-positivist view on context *via* a constructivist perspective and stronger methodological guidance will support improved use of contextual analysis for all subsequent phases of IS projects. Therefore, this paper's aims are threefold. First, we will note current conceptualizations of context and reflect on limitations of current approaches for studying context. Second, we will describe how IS methodology can be strengthened by endorsing a constructivist perspective using COVID-19 vaccination program implementation as an example. Third, we will stimulate a discussion on the methodological and practical implications on contextual analyses that endorse a constructivist perspective.

## Materials and methods

First, to identify the concepts and theoretical foundations of context in IS we conducted a narrative review of articles in electronic data bases including *PubMed, EMBASE* and *Web of Science*. We searched literature beginning with articles published in 2019, using a key word search and MeSH terms for context and implementation science (Supplementary Table 1). Gray literature was excluded. Backward searches of identified papers' reference lists led to related IS studies. We included all studies that conceptualized context or reported on its characteristics and/or reported approaches to study context as part of implementation research. Using an inductive approach, we identified and mapped ten characteristics of context (i.e., multi-dimensional, multi-level, interactional, relational, situational, constructed, sentinent, multiple, tied to meaning, dynamic).

Second, based on the characteristics identified, we selected social science concepts we deemed useful to address current gaps in approaches to contextual analysis (e.g., limited consideration of social interactions). This process was guided by the last author, who is a medical anthropologist (SS). Important sources regarding social sciences were based on recommendations of the last author (SS) and include Emirbayer and Mische (29), Cresswell (30), Cresswell (31), Lefebvre and Nicholson-Smith (32), Bourdieu (33), Bourdieu (34), Massey (35), and May (36), May and Finch (37).

## Results

### Conceptualization of context based on the context and implementation of complex interventions framework

Given the high variability of terminology and conceptualization concerning context across IS literature, TMFs, combined with a lack of well-worked-out methodology, concept analyses and other research suggest that the concept of context in IS is only partially mature (38–41).

One IS framework that gives most attention to context and emphasizes its ecological perspective—i.e., it views context as multi-level, multi-dimensional and interactive—is the *Context and Implementation of Complex Interventions (CICI) framework* (4). CICI is a meta-theoretical (determinant and evaluation) framework derived from empirical evidence. Based on a concept analysis, the CICI authors define context as an overarching concept that includes both, the setting (i.e., the physical location in which an intervention will be implemented) and broader multilevel characteristics, i.e., “roles, interactions and relationships” (4). More specifically, context refers to

“a set of characteristics and circumstances that consist of active and unique factors, within which the implementation is embedded. As such, context is not a backdrop for implementation, but interacts, influences, modifies and facilitates or constrains the intervention and its implementation” (4).

Context is multi-level (micro-, meso-, macro-level) and extends across seven domains (geographical, epidemiological, socio-cultural, socio-economic, political, legal, and ethical), each of which includes a unique set of contextual factors. Setting, which is one aspect of context, is often confused with context or used as a proxy (38, 41). However, setting can be differentiated from the broader context. It focuses on physical characteristics, work environment and practice patterns and should provide a more granular depiction of the physical location in which an intervention will be delivered (e.g., ward, hospital, country) (4). Within the setting, context interacts with the intervention and implementation (i.e., implementation theory, processes, strategies, outcomes and agents) over time (4). However, current IS TMFs afford minor importance on the differentiation between context and setting and provide little or no guidance on how to operationalize setting. Also, within CICI, the concept of setting needs further elaboration. Additionally, more guidance is required to examine poorly addressed aspects, i.e., interactions among contextual factors and across contextual levels, as well as changes in context over time.

### Current approaches to contextual analysis

Although context's importance to implementation has been emphasized, only a minority of IS studies show thorough contextual analyses, i.e., including a theoretical underpinning, using empirical evidence to identify relevant contextual factors, involving stakeholders, reporting how contextual finding inform further study phases of IS projects (e.g., intervention development/adaptation, selection of implementation strategies) (42–44). This might reflect the fact that contextual analysis tends to be poorly described, and a huge variability in methodological approaches is applied to it (26, 45, 46). In their systematic review of 64 empirical implementation studies, Rogers et al. (26) identified over 40 various measures that were applied to assess context, including TMFs and measurement tools such as the Alberta Context Measure. More than half of the studies reported the use of qualitative methods (e.g., interview methods) whereas 28 % of the studies within the review applied quantitative methods (e.g., surveys) and only 19% applied mixed-methods approaches (26). Although, quantitative results may allow for greater generalizability, they typically allow for a less rich and complex understanding of the context than qualitative or mixed methods approaches.

Further, contextual analyses are commonly neither theory-based nor linked to further study phases (42). To be clear, many TMFs do address context (e.g., the Consolidated Framework for Implementation Research (CIFR) (47) or the integrated-Promoting Action on Research Implementation in Health Services (i-PARIHS) framework (48)). However, they do not provide concrete descriptions of how to assess their specific contextual constructs.

In addition, there is enormous variability regarding which aspects of context are considered and most studies fail to convey a dynamic and interactive understanding of context including social processes (26, 42). Contextual analyses usually focus on what people say or what they say they do (e.g., their comments on resource availability, practice patterns, or readiness for change); however, they rarely observe and assess what people actually do in daily practice. Instead, they tend to focus on distinct characteristics of context that can be measured and controlled (e.g., resources, leadership) or setting factors (e.g., work processes or study site characteristics) (2, 17, 49). Rather than building an understanding of their complex context, they tend to quantify and generalize implementation determinants (i.e., assess the influence of X on Y) that affect implementation.

This linear-thinking, mechanistic approach is based on a post-positivist understanding—one that is also reflected in the IS frameworks currently available to guide contextual analysis (50).

### Limitations of contextual analysis grounded in a post-positivist perspective

To understand the interplay of factors within a given context, then to apply that understanding to IS projects' later phases (e.g., intervention development/adaptation, selection of implementation strategies, interpretation of outcomes), the post-positivist perspective exploits only a fraction of a contextual analysis' potential. This limited, post-positivist view's potential consequences become very clear in light of implementation challenges during the current COVID-19 pandemic. Many implementation strategies failed to achieve maximum potency (e.g., COVID-19 vaccination rates remain low despite incentives) or even lead to counter-intuitive effects (e.g., nudging using restrictions on other areas of public life, which may have led to increased anti-COVID-19 vaccination sentiment) (51, 52). This was among other because of choosing a one-size-fits-all approach instead of tailoring implementation strategies to context specific needs (e.g., in rural or urban areas, between different social groups). On common result is that, while some aspects of context seem favorable, implementations fail or cannot be sustained (53). I.e., although sufficient doses of COVID-19-vaccine are now available, regulatory frameworks in place, and the infrastructure prepared (vaccination centers, primary care practices, mobile vaccination teams), vaccination coverage is increasing only slowly in some industrialized countries. Why then, with easy access to safe, highly-effective vaccines, are large numbers of people not yet vaccinated? Research shows that reasons are complex and influenced by social processes in the context (54). This highlights the need in addition to focusing on the quantifiable aspects of context, refer to the dynamic nature of context and the need to explore these interactions, i.e., to understand which structures, individual views, values, and motivations underlie the agents' actions and can influence a successful implementation (16).

### Embracing a constructivist perspective regarding contextual analysis

The constructivist paradigm acknowledges the dynamic nature of context, as well as the presence of multiple, subjective realities based on individuals' lived experiences and constructed through interactions with others (Table 1) [(27), p.60]. Interacting with and within a system such as a social group or an organization (e.g., a hospital), agents tacitly agree on ideas, norms and rules that shape their actions. These same unspoken agreements make it clear when someone not strictly adheres to these norms. Since they are not verbalized and communicated actively, but adopted based on habitual everyday practices in specific settings, norms are not typically obvious to individuals. In hospitals, e.g., an unwritten rule is that only healthcare professionals can measure and record the blood pressure of chronically ill patients with hypertension (55). However, when implementing a self-management intervention that makes patients responsible for taking and recording their own blood pressure, this might affect for example healthcare professionals‘ openness regarding the intervention. Therefore, understanding this unwritten rule is important that strategies can be developed to overcome it.

**Table 1:** Comparison of post-positivist and constructivist perspectives in regard to contextual analysis in implementation science (27, 116).

	**Post-positivist perspective**	**Constructivist perspective**
Interpretive framework
Possible researcher goals	To discover potential facilitators and barriers which might impact the implementation of an intervention	To understand the complex context and setting in which the intervention will be implemented, including e.g., social, cultural, behavioral aspects and relationships
Potential researcher influences	Implementation researcher has training in quantitative and/or qualitative research	Implementation researcher has training in ethnographic methods
Examples of researcher practice	To ensure rigor, facilitators and barriers to intervention implementation are systematically assessed and analyzed	(Multiple) realities constructed by agents are interpreted by the research team
Philosophical questions
Ontology *(What is the nature of reality?)*	There exists a single, generalizable reality: Implementation of the intervention is affected by identified facilitators and barriers	Based on their lived experiences and interactions with other individuals, multiple realities are constructed by agents in view of the intervention
Epistemology *(What is the relationship between the researcher and that being researched?)*	Relevant facilitators and barriers to implementation are objectively assessed using instruments and structured assessments	The implementation researcher collects subjective information in collaboration with agents (co-construction)
Axiology *(What is the role of values?)*	Implementation researcher bias are minimized e.g., by using validated measurement scales	Implementation researcher uses personal interpretation, individual values of agents are desirable
Methodology *(What is the process of research?)*	Deductive methods are applied, e.g., testing hypotheses or theories; results are compared among participants	Inductive methods are applied, i.e., based on agents' perspectives, patterns, theories and interpretations are built up

Integrating a constructivist perspective in contextual analyses offers an additional source of knowledge: by helping to open the ‘black box‘of the context in which individuals act and interact, it also illustrates their social relationships, and how context shapes their behavior and actions in day-to-day practice and vice-versa (56–58). Via a knowledge of that underlying structure, researchers can identify and describe values and beliefs and to track their evolution over time. Within that specific context, this allows them to expose potential problems and increases their understanding of why and how this context influences implementation success (14, 50, 59).

Building on the current state of IS research, we identified five relevant concepts from sociology and social anthropology, i.e., 1) *social place and* 2) *social space*, 3) *agency*, 4) *sensation, and* 5) *embodiment*. Below, we explain each concept in a separate paragraph, indicating how it can enrich the current view of contextual analysis, thereby strengthening the basis for all later phases of the implementation project. However, as these five concepts overlap with and influence one another, they cannot be considered independently.

To illustrate the individual concepts, we used the CICI framework as a starting point, then expanded it to encompass our five concepts (4). The CICI framework focuses specifically on the context and the complex interplay between its multi-level elements whereas other well-known frameworks are less detailed in their attention to context (e.g., CFIR). We also reported on implementation challenges of COVID-19 vaccine programs to reflect several types of insights and ways in which a constructivist perspective could have helped public health officials anticipate and avoid certain problems regarding vaccination uptake (Table 2).

**Table 2:** Overview of concepts to integrate a constructivist perspective in contextual analysis exemplified by COVID-19 vaccination uptake and questions that can be applied to inform subsequent phases of an implementation science project.

**Concept**	**Definition**	**Example COVID-19 vaccination uptake**	**Questions informing next phases of an implementation science project**
Place			
Location	Defines where a place is (e.g., indicated with coordinates).	The vaccination centers are centralized in larger cities.	What is the exact location of the setting in which the intervention will be implemented? How does the location impact, for example, the reach of agents?
Locale	Physical and social aspects of a place in which social relations unfold.	Availability of public transport to access the vaccination center.	Which aspects of the setting influence the agents' actions? What physical and social resources are available in a setting that can support implementation? What other resources might be needed?
Sense of place	Individual or shared meanings or emotions associated with a place.	Primary care physician offices are associated with trusting relationships.	How do individual meanings of a setting influence agents' actions in terms of the intervention?
Social space	Social space is produced by interactions of agents, depended, e.g., of social status or economic capital.	Individuals with different social and cultural backgrounds share a common space.	Which networks of agents exist, how do they interact in daily practice (e.g., team dynamics) and what might be their potential influence on the implementation process? How can these networks or agents within them be involved within the implementation project?
Agency	Capacity of agents to shape the context in which they are situated at a given point in time based on their experience, personality, knowledge, skills, beliefs, attitudes or their structural social position.	Given their trustworthiness, religious leaders can exert a considerable influence on members of their community to get vaccinated.	Who are agents that are important to the implementation of an intervention? Depending on the intervention to be implemented, they can be on different levels, e.g., individuals, communities, organizations. Which agents have a higher level of agency and might act as gatekeepers for implementation? Which implementation strategies will be appropriate to enhance the agency of agents to support implementation in practice?
Sensation and embodiment	Lived experiences agents perceive with their bodies in social and ecological contexts, that shape their actions.	The place where the vaccines are administered makes individuals feel uncomfortable.	How do embodied experiences of agents shapes their action, e.g., to adopt an intervention? Which intervention components or implementation strategies are more appropriate for these agents?

#### Place and social space

Within the concept of context, place refers to the physical setting, whereas social space represents the abstract dimension in which relationships and interactions of individual agents occur (31). The concept of *place* helps to operationalize the setting. It combines the three elements of *location, locale, and sense of place* (30). Location and locale are usually assessed by default in IS.

##### Location

According to Creswell (2014), location is an ‘absolute point in space‘, which has a certain distance from other locations, i.e., it defines where a place is (e.g., via coordinates) (31). Characteristics of the location can affect agents‘ behavior. E.g., location can refer to the individual vaccination centers that administer the COVID-19 vaccination. At the beginning of the pandemic, most of these were centralized in larger cities. While they were easily reachable by individuals living nearby or using public transport, those with limited mobility or in rural areas struggled, e.g., to access the vaccination center or were burdened by travel expenses. These factors limited the utilization of such centers (60). When establishing vaccine distribution networks and supply chains, particularly in areas where decentralized centers later emerged, the distance between vaccination centers affected both the supply and uptake of vaccines (61).

##### Locale

Locale includes a combination of physical and social aspects in which individuals' social relations unfold (30, 31). Physical aspects refer to “the landscape of a place—its physical manifestation as a unique assemblage of buildings, parks, roads and infrastructure” (31). Social aspects identify a locale as “a setting for particular practices that mark it out from other places” (31). In our example, physical aspects can refer to the physical existence of buildings or vacant land where vaccination centers can be established. They also include available infrastructure, e.g., public transport, that allows individuals to access the centers or otherwise supports vaccine uptake. Social aspects of locale include places that are well-frequented during pandemic times, such as supermarket parking lots. The deployment of mobile vaccination teams in these areas offers an efficient way to increase vaccinations' reach and adoption.

##### Sense of place

While locale refers to the observable and tangible aspects of a place and its uses, sense of place refers to its subjective aspects. These include the meanings individuals or groups associate with a place, particularly the feelings and emotions it evokes (30, 31). For example, many people think of their primary care physicians' offices as places where they can go with all of their health concerns. This perception is based on long-standing trustful relationship with their primary care providers. For such people, receiving vaccinations from their primary care physicians rather than from healthcare professionals in a vaccination center can enhance vaccine uptake (62, 63).

For contextual analysis, the concept of place helps clarify our understanding of setting and context, and to specify aspects of a setting that require analysis. Exploring place in implementation studies will foster an understanding of the structures, values, beliefs and shared meanings, feelings or emotions that affect agents' actions. In particular, understanding the sense of place agents associate with a particular locale will add a useful perspective. This will both enhance the granularity of the IS researchers' contextual data and deepen their understanding of which aspects of a setting influence agents' action.

For COVID-19 vaccination campaigns, the concept of place could inform multiple implementation strategies to enhance and address barriers to vaccine uptake. These strategies could include, e.g., providing free transportation to vaccination centers for low-income individuals to overcome cost barriers; and for those with limited mobility, either special-needs transportation, offering vaccinations in high-traffic, easily-accessible areas (64).

##### Social space

The concept of social space implies multilevel interactions driven by characteristics of place and social relationships between agents (65). Exploring social spaces informs our understanding of how social interactions influence agents' decisions and behaviors in practice (65, 66). Social space is never static; it is continuously shaped and reshaped through lived experience of everyday practices [(35), p.283, (67)]. Social space depends on social milieus and on agents' positions within their society. Societal positioning results from interactions between the specific rules of the field (a setting in which agents and their social positions are located), each agent's habitus (ingrained habits, skills and dispositions) and each agent's social, economic and cultural capital (34, 68). Between individual agents within a group, strong boundaries can exist (68). The more closely agents, groups or organizations are located within a space, the more properties they will have in common (33, 68). Social spaces exist across national borders or within societies, families, workspaces, or cities (69).

During the COVID-19 pandemic, vaccination hesitancy varies considerably among social groups that share common spaces, e.g., younger individuals, low-income communities, rural residents, or migrant populations (70, 71). E.g., younger adults' concerns vary from those in other social groups as their doubts about the safety or side/adverse effects of vaccines may focus more on fertility/pregnancy (60). Addressing such concerns and enhancing vaccine uptake will require targeted education, outreach programs or mass media disseminated *via* channels popular among younger adults (e.g., social media platforms and internet) (71). Also, very well-networked individuals can act effectively as role models/influencers. Being aware of such central roles can be extremely useful, e.g., for improving communication processes or facilitating implementation.

As part of context, social space influences context and daily practice routines and helps to explain changes in both (72). To understand social space, it is important first to know the place, affiliations, relationships between agents, including their relative power, social backgrounds including culture, and economic capital (66, 68). It is important to identify agents that share a social space as this space impacts their decisions and behaviors (66). Each space includes its own combination of implementation-relevant factors, any of which might influence a proposed implementation strategy's effectiveness. Therefore, an awareness of a context's main social spaces might help to improve the fit of implementation strategies.

#### Agency

Agency is an important feature of context and refers to agents' capacity to shape the context in which they are situated at a given moment (29). Agents can be individuals, groups or organizations within a context, who respond interactively and dynamically to changes in the context. These responses depend on their past experiences (habits), their underlying mental models (e.g., norms, attitudes), and their structural social positions (29, 73?, 74).

Implementation processes depend on agency, whereas variations or changes in context (e.g., an intervention' implementation) affect individuals' agency (37). Thus, within IS projects, it is important to understand how the various agents are embedded within their context, how their actions affect the context and, based on that, what value each agent can add to a successful implementation (73). Some agents are assumed to have a higher level of agency. In Rogers' theory of diffusion, these are known as innovators and early adopters (75). Other agents, e.g., the late majority, have lower levels of agency (73). Power structures (e.g., team hierarchy, dominant roles), the belonging to specific social groups (e.g., depending on age or gender), and variations or changes in context (e.g., social, cultural, economic, relational) affect individuals' agency (e.g., in decision-making processes) and impact on intervention implementation (e.g., senior physician support for an intervention leads to increased adoption among other physicians) [(37, 76), p.29–30, (77)].

Regarding COVID vaccine uptake, agency becomes apparent not only when accepting but also when refusing vaccination, e.g., because of religious reasons, beliefs, attitudes with healthcare practice, distrust of government (54, 60). Also individual worldviews, such as the neoliberal belief that each person is individually responsible for his or her health—in contrast to a collective responsibility—affect vaccine uptake (78). However, some factors might also shape individuals' agency. For example, education, language skills or health literacy might all affect access to healthcare services, influence the agent's ability to detect misinformation or interpret conflicting or changing information (60, 79, 80). Other individuals perceived as powerful might act as a ‘gatekeeper for implementation', e.g., encouraging others to get vaccinated. These are often religious leaders, specific family members, or community leaders (60).

However, as noted, besides responding to context, agents can change context either through acting or through refraining from action. For example, after vaccination certificates became necessary to enter restaurants some individuals counterfeited them or banded together in political groups to stoke anti-vaccine beliefs (81). When choosing implementation strategies to increase vaccine uptake, such capacities need to be considered.

Relative to contextual analysis as a whole, the concept of agency acknowledges not only how agents mutually constitute and influence one another, but also how they interact with their context (29, 74?). Early identification of agents with high levels of agency will help implementation scientists facilitate the adoption and sustainability of their target interventions. Specific implementation strategies are also available to enlist agents with lower levels of agency [e.g., providing multilingual reading or mass media campaigning for those with limited language skills (64)].

#### Sensation and embodiment

In IS, the concepts sensation and embodiment have often gone unnoticed. Embodiment reflects the lived experiences—those agents perceive directly *via* their corporal and lived bodies in social and ecological contexts—that shape agents' actions, and thus also, whether they choose to support interventions' adoption and implementation [(82), p.28, (83–85)].

The corporal body can be distinguished from the lived body (86). Whereas the corporal body is substantive and measurable (i.e., it has a mass, occupies space, and performs diverse physical functions), the lived body refers to the subjective, lived experience based on sensation, i.e., “touch, proprioceptive sensations, kinesthetic sensations” (87). Thus, as it relates to contextual analysis, the body can be viewed as a tangible resource that produces outputs, but that also embodies lived experiences agents gain throughout their daily lives (e.g., stress, burnout, discrimination) and affecting their actions (83, 88, 89). E.g., members of marginalized social groups, whose experiences of social exclusion have eroded their trust in government and, by extension, vaccines (78). Other individuals may habitually express those lived experiences in everyday life, in “gestures, tone of voice, emotions, body posture, bodily contact and language” (90). For example, individuals with pre-existing conditions or who are concerned about contracting COVID for other reasons unconsciously stay further away from people who may not be vaccinated.

Both sensation and embodiment are essential to human agency. After COVID−19 vaccines were widely available, it quickly became apparent that young healthy people, or those with few healthcare contacts, perceived their risk for severe COVID infection as very low and thus refused vaccination (91–93). Some, having experienced side effects from their first dose, refused a second dose or booster (94, 95). Also, when thinking about the setting where vaccines are administered, some people might feel uncomfortable when being processed through a vaccination center, others might be overwhelmed at being treated by care staff they have never met. Whether positive or negative, underlying experiences and attitudes are implicit in individuals' behavior and can influence the implementation (54). Considering embodiment within an implementation context and helping agents articulate their lived experiences facilitates understanding of the contextual mechanisms that shape agents' actions, while exposing leverage points for contextually adapted implementation strategies (e.g., tailored measures for increasing marginalized groups' trust in vaccines) (96).

### Methodological implications: Use of rapid qualitative methods and rapid ethnographies

Studying context from a constructivist perspective requires additional methodological considerations for data collection, data analysis and reporting.

#### Data collection

To qualitatively study context, most commonly interview methods are used (26). However, as not all participants are equally available for the interviews or express themselves openly, interviews only provide initial qualitative information, e.g., about participants' levels of agency, their relationships, mental models and expectations (69). Therefore, using a range of qualitative methods including various forms of interviews, direct observation, document analysis, or case study approaches is recommended (24, 69, 97).

Observations have the potential to provide a holistic view by exploring the agents' processes (implicit or habitual), interactions and behaviors that might otherwise be considered commonplace or unintentional, or simply not accepted, leaving them unaddressed (98, 99). Further, informal knowledge, shared formal practice as well as mismatches between recommended practice and actual practice can be uncovered (57). Alongside observation, document or archival analysis can be utilized to develop an understanding of historical or policy-related context influencing agents' actions (98). To support ongoing assessment of context, e.g., interviews with key agents ranging from informal conversations to semi-structured interviews can be conducted (100). Additionally, fieldnotes can be taken to record changes in context, e.g., during regular team meetings or discussions with key agents (98).

Empirical case study approaches, are another method, that is particularly suited to study in-depth dynamic interactions in context and incorporates various sources of information such as those mentioned above [27, p.153, 98]. Case studies are defined as “qualitative approach[es] in which the investigator explores real-life, contemporary system (a case) or multiple bounded systems (cases) over time, through detailed, in-depth data collection” [27, p.153]. Case study approaches cannot only be applied to study context before the start of an implementation science project (e.g., to inform for intervention development), but also to evaluate complex interventions (97).

Case studies are often conducted within ethnographic approaches, which seem to be well-suited to study the complex and dynamic interactions between context, implementation and intervention (56, 101, 102). Ethnography is a theory-driven approach, providing a detailed description of diverse agents, their behaviors and interactions in everyday practice, as well as how agents make sense of the context based on their norms, values, beliefs and roles [27, p.148, 102]. In comparison to other qualitative approaches, ethnography specializes in the study of larger groups of agents interacting over time. This suits it well to multisite research, which might be interesting for larger IS projects targeting multiple settings [27, p.143, 102].

However, for contextual analysis, qualitative and particularly ethnographic approaches have been criticized as costly and time consuming, generating large volumes of data (2). As the resources for a contextual analysis are usually limited, we argue that to inform later phases of an implementation project optimally, it is important to generate as much information as our resources will allow. This is especially true for the COVID-19 situation, where in a relatively short time, a comprehensive understanding of context had to be acquired. This need has led to the current focus on rapid qualitative and ethnographic methods within contextual analysis (2, 103). E.g., rapid ethnographies exploit diverse sources including interviews, observations, focus groups and mapping processes (104). Those methods allow researchers to reliably, efficiently and affordably gather more contextual information in a shorter time (105). Besides that, rapid ethnography and elements of rapid evaluations (e.g., advisory boards, feedback loops) have been combined to allow longitudinal assessments of context and observation of changes over time [(106), p. 41].

However, in addition to strong familiarity with the methods themselves, applying them requires at least a basic understanding of the setting in which an intervention will be implemented. Otherwise, a deep-dive contextual analysis is needed.

#### Analysis and reporting

Context is situational and continuously shaped and reshaped. Constructed by various agents, its characteristics depend on situational aspects and prevailing conditions at the time of observation (107). To recognize changes in context and enable adaptions of intervention and implementation strategies to fit the evolving context, context should be assessed longitudinally (108). For instance, regular stakeholder meetings or informal exchanges with agents can highlight early signs of changes that require adaption. However in-depth up-to-date contextual knowledge is acquired, it is an essential prerequisite to addressing contextual changes in ways that sustain interventions in daily practice (12). When reporting the findings of a contextual analysis, in addition to descriptive data and narratives, case studies, vignettes or typologies can be employed.

### Implications for practice: Considering the position of the researcher studying context

Considering the five constructivist concepts provides a rigorous way to increase understanding of complex and dynamic interactions in context. These insights allow researchers to identify practices or aspects that might impact their intervention and implementation processes, as well as key agents that need to be closely monitored throughout the implementation process (102, 109). The findings gained from a contextual analysis are particularly dependent on who is conducting the contextual analysis, from what perspective, and whose context is being studied. As such, different kinds of context(s) might exist for different individuals, i.e., contextual findings might differ for example between physicians and nurses. But also the perspective (e.g., “insider—outsider position”) of the researcher studying context and the researcher‘s conceptualization and operationalization of context (e.g., as multi-dimensional, multi-level and dynamic) will affect the research questions and findings of a contextual analysis. According to Meier & Dopson [83, p.16] the three main questions in contextual analyses entail 1) What constitutes the context of a situation/event or phenomenon?; 2) How do actors understand, experience and engage with context in a given situation or phenomenon?; and 3) How do contexts change, and what is the role of actors in such process? For example, implementation researchers may actually be part of the setting (“insider position”), i.e., they may be analyzing part of an academic institution with which they are associated [cf. embedded implementation research (110, 111)]. Compared to external observers (“outsider position”), these researchers start with inside knowledge of the context and setting. This will give them a different perspective during the contextual analysis, i.e., internal analysts will focus on different contextual factors than their external counterparts.

In fact, using embedded researchers to perform contextual analysis is recognized as an implementation strategy in itself. One advantage such researchers offer is that, if they have experience from previous projects in the same setting, they will likely have a working knowledge of the structures, processes, practice patterns and culture. By helping them to focus on relevant contextual factors, such knowledge helps them first select target factors, then conduct their contextual analysis. It also supports the involvement of agents within the setting, and may even promote the proposed intervention's implementation and sustainability (111).

One obvious risk is that notable choices (e.g., of intervention components and implementation strategies) will be based on implicit knowledge, making them intransparent to external researchers. Moreover, experience within a setting shape researchers' observations both of context and of setting (e.g., confirmation bias), thereby influencing their findings and conclusions. Therefore, in addition to ensuring that the perspectives they consider are representative of all agents within the setting, embedded researchers need to reflect carefully on their own positions and how this might affect how they interpret their findings (111). In contrast, external researchers or practitioners conducting a contextual analysis must first develop a working understanding of how the context works. Particularly for those researchers, taking a constructivist perspective will increase the depth of their contextual analysis and help to make otherwise invisible aspects of context visible.

## Discussion

**Figure 1:**
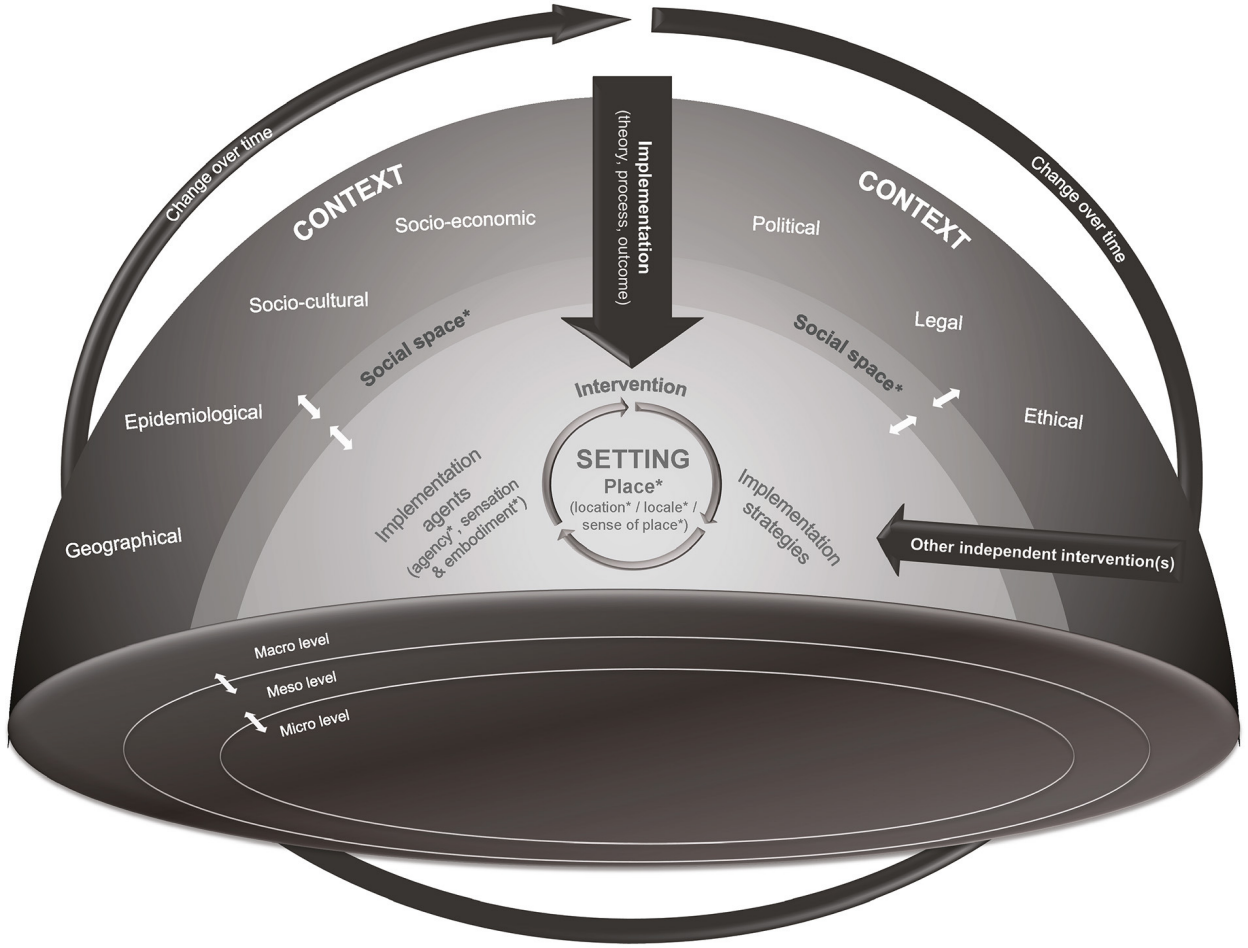
Adapted CICI framework (4) based on identified concepts (marked with *). Context is multidimensional, including seven context domains: geographical, epidemiological, socio-cultural, socio-economic, political, legal, ethical) and multilevel (micro-, meso-, and macro-level). Within a context several social spaces exist, providing a condition for social interactions between agents that shape context and setting. Setting is intertwined with context and combines the three perspectives of place: location, locale and sense of place. The implementation (including implementation theory, process and outcomes) of an intervention takes place in the setting. During and after implementation, the intervention interacts with implemented implementation strategies and agents or other independent interventions implemented at the same time in the setting. Those interactions mutually shape and reshape the context. Agents have the capacity (agency) to change context and setting, but also to respond to changes in context and setting. Thus, context is situational, dynamic and continually evolving. This figure has been adapted from Pfadenhauer et al. (4) with the permission of the author Lisa Pfadenhauer.

By enriching contextual analysis from a constructivist perspective, this paper promotes a multi-layered approach to contextual analysis and complements previous conceptualizations of context (Figure 1). Based on this, we understand context as an overarching, multidimensional, multilevel concept. It consists of a set of interrelated characteristics and patterns, and is both enabled and driven by its various agents' social structures and underlying values and beliefs. Within a context, multiple social spaces generally exist. These are essential for social interactions between the agents who shape the context. Intertwined with context, setting combines the three perspectives of place: location, locale and sense of place. The setting is where an intervention is implemented and where it then interacts with implemented implementation strategies, agents and/or any concurrent interventions. Those interactions mutually shape and reshape the context. Agents have the capacity to change context (agency), but also to respond to its changes. Thus, context is situational, dynamic and continuously evolving.

For research teams conducting contextual analyses, a constructivist perspective, as compared to a post-positivist perspective, enables a more detailed view of context and reveals complex dynamics. In addition, realist approaches have become more popular in IS as they account for context as well. However, the constructivist perspective exceeds that of the realist methodology (112). While the realist methodology focuses on context-mechanism-outcome configurations to understand what needs to happen for a successful implementation, a constructivist perspective helps to understand agents' actions regarding implementation (24, 113–115). To understand what agents do, it is necessary first to identify the social structures, norms, values and beliefs that drive their actions, and to explore how context and agents interact and mutually influence one another (58). Thus, we hope that this paper contributes to a stronger conceptualization of context. And finally, we strongly believe that approaching contextual analysis from a constructivist viewpoint broadens and deepens the contextual knowledge available to inform IS projects' subsequent phases, thereby maximizing both uptake and sustainability (8, 9, 52, 109).

## Data availability statement

The original contributions presented in the study are included in the article/supplementary material, further inquiries can be directed to the corresponding author.

## Author contributions

JM, SS, and SD conceptualized the manuscript. JM drafted the manuscript. SS provided additional text to the draft manuscript. SS, SD, FZ, LP, and TB provided critical feedback and revised several iterations of the manuscript. All authors read and approved the final manuscript.

## Conflict of interest

SD consults for Sanofi and Novartis. LZ declares research funding awarded from Proteus Digital Health and the PhRMA Foundation, and consults for Novartis and Pfizer. All activities are unrelated to the current work. The remaining authors declare that the research was conducted in the absence of any commercial or financial relationships that could be construed as a potential conflict of interest.

## Publisher's note

All claims expressed in this article are solely those of the authors and do not necessarily represent those of their affiliated organizations, or those of the publisher, the editors and the reviewers. Any product that may be evaluated in this article, or claim that may be made by its manufacturer, is not guaranteed or endorsed by the publisher.

## Abbreviations

CICI framework: Context and Implementation of Complex Interventions (CICI) framework
IS: implementation science
TMF, theory: model or framework.
