# Supplementary material for: Understanding dynamic complexity in context—Enriching contextual analysis in implementation science from a constructivist perspective
Source: Front Health Serv. 2022 Jul 22;2:953731. doi: 10.3389/frhs.2022.953731 (PMC10012673; doi:10.3389/frhs.2022.953731)
Supplement: Supplementary file 1 [file Table_1.DOCX]

Supplementary Material

# Supplementary Table 1

| **Search string PubMed** |
| --- |
| contextual analysis[Title/Abstract] OR context[Title/Abstract]) OR setting[Title/Abstract] OR environment[Title/Abstract] AND implementation science[Title/Abstract] OR implementation science[MeSH Terms] OR implementation research[Title/Abstract] OR improvement science[Title/Abstract] OR knowledge translation[Title/Abstract] OR knowledge to action[Title/Abstract] OR dissemination science[Title/Abstract] OR translation science[Title/Abstract] |
| **Search string EMBASE** |
| 'context'/exp OR 'context' OR 'context* analysis' OR setting OR 'environment'/exp OR 'environment' AND 'implementation science'/exp OR 'implementation science' OR 'improvement science' OR 'knowledge translation'/exp OR 'knowledge translation' OR 'knowledge transfer'/exp OR 'knowledge transfer' OR 'dissemination'/exp OR 'dissemination' OR 'translational research'/exp |
| **Search String Web of Science** |
| TS=(context) OR TS=(context* analysis) OR TS=(setting) OR TS=(environment) AND TS=(implementation science) OR TS=(implementation research) OR TS=(improvement science) OR TS=(knowledge translation) OR TS=(knowledge to action) OR TS=(dissemination science) OR TS=(translation science) |
